# Attitudes and referral practices for pre-exposure prophylaxis (PrEP) among HIV rapid testers and case managers in Philadelphia: A mixed methods study

**DOI:** 10.1371/journal.pone.0223486

**Published:** 2019-10-07

**Authors:** Iman Kundu, Ana Martinez-Donate, Navya Karkada, Alexis Roth, Marisa Felsher, Marcus Sandling, Zsofia Szep

**Affiliations:** 1 Department of Environmental and Occupational Health, Drexel University Dornsife School of Public Health, Philadelphia, PA, United States of America; 2 Department of Community Health and Prevention, Drexel University Dornsife School of Public Health, Philadelphia, PA, United States of America; 3 Department of Medicine, Division of Infectious Diseases & HIV Medicine, Drexel University College of Medicine, Philadelphia, PA, United States of America; Hunter College, UNITED STATES

## Abstract

**Objective:**

Adoption of pre-exposure prophylaxis (PrEP) to prevent HIV infection has been slow. The purpose of this study was to evaluate knowledge, attitudes and referral practices for PrEP among non-prescribing providers, who may play key role.

**Methods:**

We performed a cross-sectional survey on PrEP knowledge, attitudes, and referral practices among 66 non-prescribing HIV prevention providers (1st August to 31st December, 2016), in Philadelphia, followed by qualitative interviews with 12 of them (5th April to 10th May, 2017).

**Results:**

Participants had a mean age of 36 years, with 62% females. Majority were HIV case managers and rapid testers. For half of the respondents, PrEP eligibility screening was part of rapid HIV testing at their organization, 40% never had PrEP training and only 27% indicated personally screening clients for eligibility. Qualitative data revealed that participants held positive attitudes about PrEP and perceived organizational support, but had concerns about potential negative impacts and barriers to routine HIV screening.

**Conclusion:**

Results highlight the importance of training non-prescribing HIV prevention providers about PrEP, addressing their concerns, and incorporating PrEP screening and referral into routine HIV testing.

## Introduction

Pre-exposure prophylaxis (PrEP) is a biomedical HIV prevention strategy approved by the U.S. Food and Drug Administration (FDA) in 2012 [1]. It allows individuals at risk for HIV *to prevent* the infection by taking an anti-retroviral medication, emtricitabine/tenofovir, on a daily basis [2]. In clinical trials, PrEP has been shown to reduce the risk of acquiring HIV by 44% among men who have sex with men (MSM), 75% among heterosexuals, and 49% among intravenous drug users [3,4,5]. In two real world studies, PrEP was shown to reduce the risk of HIV acquisition by 86% [6,7]. Despite the approval of PrEP by FDA and the potential of this biomedical prevention method to help control the HIV epidemic, the number of HIV diagnoses in the U.S. decreased only from13.5 per 100,000 in 2011 to 11.8 per 100,000 in 2017 [8,9]. One reason for the slow decline in HIV diagnoses may be the low uptake of PrEP in real-world clinical settings, particularly among at-risk women and racial/ethnic minorities [10,11].

Research to identify individual-, provider-, and structural-level barriers for PrEP uptake and adherence among at-risk populations is imperative if we are to reap the benefits of this new prevention method. An analysis of 2014 National HIV Behavioral Surveillance data showed that although more than 50% of MSM were eligible for and willing to take PrEP, only 4% reported using PrEP [12]. Similarly, a recent study with 995 HIV negative gay and bisexual men in the United States showed that 65% were eligible for PrEP, but only 4% were on PrEP [13]. Qualitative research has shown that PrEP is an attractive prevention option for women, but most are unaware of PrEP and few have been told about PrEP by their providers [14].

Much of the research to date on providers’ role in facilitating or impeding widespread PrEP adoption has focused on physicians and other prescribers. A 2014 study of Canadian physicians showed that the participants had concerns regarding cost, efficacy and implementation of PrEP and 3 out of 4 providers thought they did not have enough information on PrEP [15]. A survey of US family planning providers found that less than 50% of the respondents answered PrEP knowledge questions correctly and authors identified the lack of provider training as the main barrier for PrEP prescription [16]. There is also an uneven distribution of knowledge, attitude and practice regarding PrEP among providers. HIV specialists have been found to have greater awareness about PrEP and are more comfortable prescribing it, compared to general physicians [17].

As with providers like physicians and nurses who are able to prescribe PrEP to eligible candidates, research into the PrEP attitudes and referral practices of “non-prescribing providers” involved in HIV prevention, is also necessary. Such providers include rapid HIV testers, case managers and navigators. Though not licensed to prescribe medication such as PrEP, they are often the first contact for clients at high risk for HIV infection and who may be PrEP eligible. Rapid HIV testers are not only in a position to determine PrEP eligibility of at-risk individuals through HIV testing and assessment of risk factors, they can also provide counselling to those who are PrEP eligible and can assist candidates who are motivated but lack a plan of action by addressing any possible concerns and/or linking them to proper PrEP services. Furthermore, their outreach to community settings can help in overcoming the barriers between at-risk individuals and PrEP services in traditional healthcare settings. Similarly, HIV case managers and navigators regularly come in contact with the partners of HIV positive individual, some of whom may be at risk for HIV and benefit from PrEP. Hence, they are in a position to intervene and counsel the PrEP eligible partners. Providers in all these roles have unique access to people who are concerned about their health, seeking HIV preventative services, and are more receptive to counselling. However, little research has been conducted to study the knowledge, attitude and referral practices of non-prescribing providers such as rapid HIV testers and case managers, who may be key to educating and referring PrEP-clients for PrEP care. A study by Senn et al (2013) which evaluated PrEP knowledge and attitudes of front-line providers, working in the roles of counselors, educators and community outreach coordinators for AIDS Support Organizations in Canada, found that providers were cautiously optimistic about PrEP and PrEP approval was associated with more years of working in HIV prevention [18]. Importantly, more than 60% participants thought they did not have adequate knowledge about PrEP. In another 2016 study based in the UK, 65% of the non-prescribing providers said that they did not have enough current knowledge for informed discussion about PrEP [19].

The primary aim of this mixed-method study was to examine demography, knowledge, attitudes and referral practices of non-prescribing providers of HIV prevention services in the City of Philadelphia, PA, a city representing the epicenter of the HIV epidemic in Pennsylvania and with a new HIV diagnoses rate almost 2.75 times higher than the national average [20]. A secondary aim was to compare knowledge and attitudes by type of non-prescribing provider. A mixed-method approach to the study will help us identify the barriers in attitude and PrEP referral practices of non-providers and then, further study to see how they are shaped by subjective norms and organization-wide practices, to be able to properly intervene.

## Methods

A mixed methods approach was utilized. The quantitative component was designed to obtain initial estimates of knowledge, attitudes and referral practices among non-prescribing providers of HIV prevention services. The qualitative component was designed to add depth, meaning, and context for these estimates and to identify additional factors not captured by the survey that may influence PrEP referral practices among these providers. Mixed methods are increasingly recommended in health research to help counterbalance the weaknesses of either approach [21].

### Quantitative approach

A cross-sectional survey of 66 non-prescribing providers was conducted between 1^st^ August and 31^st^ December, 2016. Participants were recruited from seven clinical and non-clinical HIV prevention community-based organizations (CBOs) in Philadelphia, Pennsylvania, two of which were LGBT specialty centers. Participants were included if they were non-prescribing providers of HIV prevention services, including HIV case managers, rapid HIV testers and HIV patient navigators. Medical providers who are able to prescribe PrEP were excluded from our study. Data collection was done on-site at the participating organizations during staff meetings. The purpose of the study was described to the eligible participants and consent was obtained from interested individuals.

A paper-based survey was developed based on previously published literature [15,16,22] and included 54 questions focused on four domains: socio-demographics, referral practices, knowledge and attitudes about PrEP S1 Appendix. The survey was administered to the study participants and included questions on all four domains. Knowledge was assessed using five subjective (eg. Have you ever had formal training on PrEP?) and four objective questions (eg. PrEP is a way to prevent HIV by taking HIV treatment medication after exposure to the virus.). A mean score on the knowledge scale was calculated based on the objective questions, with one point for each correct answer. The 11 attitude questions addressed both positive (eg. PrEP should be widely available based on current evidence) and negative attitudes (eg. PrEP could shift too much focus from other HIV prevention efforts) towards PrEP and consisted of dichotomous (Yes or No) responses. Five additional questions addressed PrEP-related practices at the individual (eg. Do you personally screen clients who test HIV negative for PREP?) and organizational level (eg. Is PrEP screening a part of rapid HIV testing process at your institution?).

### Qualitative approach

About half of survey participants (N = 35) was invited to complete an in-depth, face-to-face, qualitative interview between 5^th^ April and 10^th^ May, 2017. A purposive sampling strategy was used to recruit a diverse sample of 25 providers based on gender, type of providers (case managers, HIV testers and navigators), age group, educational level, and work experience. Potential participants for the qualitative study were re-contacted through phone calls and emails by research staff. Twelve participants (34% of all contacted) agreed to participate, provided written consent, completed a face-to-face interview, and were compensated with a $10 gift card. Prior to qualitative interviews, all providers had undergone a brief educational training about PrEP that gave an overview of what PrEP is, guidelines to use PrEP and research supporting PrEP. An interview guide with open-ended questions was used to obtain a better understanding of the providers’ referral practices and identify barriers and facilitators for referring their clients to PrEP services. The interview guide S2 Appendix was developed based on the information gathered through literature review, results of the quantitative study, and the Theory of Planned Behavior (TPB) [23]. The Theory of Planned Behavior has been used extensively in health promotion [24] and has proven successful to explain a wide range of health-related behaviors [25, 26, 27], including providers’ practices [28] and to develop effective sociobehavioral interventions in public health [29]. The strengths of the theory include the attention to the role of beliefs, subjective norms, and individual and environmental constraints as predictors of intentions and the emphasis on the predictive value of intentions in relation to future behaviors. With attention to a range of factors (ie. attitudes, social norms, perceptions of control, and intentions), the model offers multiple targets for the development of interventions to promote behavior change. Applied to PrEP referral practices, the TPB would posit that providers’ attitudes about PrEP; subjective norms regarding PrEP, particularly clients’, colleagues’, and organizational interest and support for PrEP; and the providers’ perceived control over their ability to refer clients to PrEP would influence providers’ behavioral intentions to refer clients to PrEP services. In turn, providers’ behavioral intentions would impact their referral practices. The interviews lasted between 45–60 minutes. All study procedures were reviewed and approved by Drexel University’s Institutional Review Board.

### Data analysis

For analysis of continuous and categorical data, standard descriptive statistics (i.e. mean, standard deviation [SD]) and percentages were calculated, respectively. For exploratory analyses comparing responses by type of provider (i.e. HIV tester vs. navigator), we used chi-square/ Fisher’s exact test with categorical variables and Mann-Whitney U test with continuous variables. STATA 13 and SAS 9.4 were used for data analysis.

All qualitative interviews were audio recorded and transcribed verbatim by one of the authors (NSK). De-identified transcripts were reviewed for quality purposes by a second research assistant and coded by one of the authors (NSK) using Dedoose (SocioCultural Research Consultants, LLC; Manhattan Beach, CA) software. A mixture of both deductive and inductive coding was used, with an initial set of primary codes based on the Theory of Planned Behavior and interview guide as well as new codes emerging from the transcripts. Primary and emergent codes were reviewed by authors NSK, AMD, and MF. The Theory of Planned Behavior was used to integrate and organize the findings. The data allowed us to achieve theoretical saturation [30]. We focus this report on examples that illustrate three main constructs of this theory: attitudes, subjective norms, and perceived control over PrEP referrals.

## Results

### Quantitative survey

#### Demographic characteristics

The sample was 62% female, with a mean age of 36 years (SD = 12 years). The majority of participants were HIV case managers (52%) and HIV rapid testers (27%). More than 80% of them served at-risk populations, including MSM, trans men and women, sex workers, and intravenous drug users (Table 1). Only 14% of survey participants worked in a setting where PrEP prescribing services was available on-site.

**Table 1:** Demographic characteristics of non-prescribing providers in Philadelphia.

Characteristic	Quantitative Survey Participants (N = 66)	Qualitative Interviews Participants (N = 12)
**Age in years**^**a**^	35.5 ± 11.9	40.0 ± 12.59
**Years in HIV prevention**^**b**^	4 (2 to 9)	8.25 (2 to 30)
**Gender (female)**	40 (61.5%)	5 (42%)
**Race**		
White	26 (39.4%)	4 (33.3%)
African-American/ Black	19 (28.8%)	4 (25%)
Hispanic/Latino	11 (16.7%)	4 (33.3%)
Asian	1 (1.5%)	0
Multi-racial	7 (10.6%)	1 (8.3%)
**First language (English)**	56 (84.6%)	9 (75%)
**Education**		
High school diploma	6 (9%)	2 (16.7%)
Some College	7 (10.6%)	1 (8.3%)
Associates College	7 (10.6%)	1 (8.3%)
Bachelors degree	19 (28.8%)	2 (16.7%)
Masters degree	26 (39.4%)	6 (50%)
**Type of provider**		
Case manager	34 (51.5%)	6 (50%)
HIV tester	18 (27.3%)	5 (41.7%)
Navigator	4 (6%)	1 (8.3%)
Others^c^	10 (15.2%)	
**Job setting**		
Community health center	20 (45.5%)	3 (33.4%)
Academic hospital	4 (9.1%)	4 (44.4%)
Walk-in clinic	4 (9.1%)	0 (0%)
Community hospital	3 (6.8%)	1 (11.1%)
Sexual health clinic	1 (2.2%)	0 (0%)
Multiple	12 (27.3%)	1 (11.1%)
**Risk group(s) serving**^**d**^		
MSM	55 (83.3%)	10 (83.3%)
Trans women/men	42 (63.6%)	10 (83.3%)
Sex workers and their clients	38 (57.6%)	7 (58.3%)
Intravenous drug users	37 (56.1%)	8 (66.7%)
Prison inmates	32 (48.5%)	3 (25%)
Heterosexual men and women	10 (15.5%)	6 (50%)

N, Number in that group; MSM, men who have sex with men.

^a^ Mean±SD, n (%)

^b^ Median (interquartile range)

^c^ Others include non-prescribing providers who are non-tester, non-case manager, non-navigator providers or providers with multiple job roles

^d^ Cumulative exceeds 100% due to multiple selections

#### Referral practices

Half of the participants reported that PrEP screening was part of the rapid HIV testing process at their organizations and 27% reported personally screening their clients for PrEP referrals. Approximately, 25% of the respondents reported having been asked about PrEP by more than five clients in previous 3 months and 16% of the providers referred more than five clients in that same period (Table 2).

**Table 2:** Current referral practices and knowledge of non-prescribing providers in Philadelphia (N = 66).

Practice	Total (N = 66)	Case managers (N = 34)	HIV testers (N = 18)	Other^c^ (N = 14)	P-value^d^
**Is PrEP screening a part of rapid HIV testing process at your institution?**					0.01
Yes	33 (50%)	12 (35.3%)	12 (70.6%)	9 (69.2%)	
No	10 (15.2%)	5 (14.7%)	4 (23.5%)	1 (7.7%)	
Don’t know	21 (31.9%)	17 (50%)	1 (5.9%)	3 (23.1%)	
**Do you personally screen clients who test HIV negative for PREP?**^**a**^ **(No)**	17 (27%)	2 (6.1%)	10 (58.8%)	5 (38.5%)	<0.01
**Is everyone who is eligible referred to PrEP services?**^**a**^ **(No)**	41 (69.5%)	17 (56.7%)	14 (82.3%)	10 (83.3%)	0.10
**How many times were you asked about PrEP by clients in the previous 3 months?** **(≤ 5)**	47 (74.6%)	28 (84.9%)	9 (52.9%)	10 (76.9%)	0.02
**How many people have you referred for PrEP in the previous 3 months? (≤ 5)**	54 (84.4%)	30 (90.9%)	11 (64.7%)	13 (92.7%)	0.05
**Subjective Knowledge Questions**					
**Have you heard of PrEP before?**^**a**^ **(% Yes)**	98	97	100	100	1.00
**Have you ever had formal training on PrEP?**^**a**^ **(% No)**	40	52.9	29.4	21.4	0.10
**How would you describe your current knowledge about PrEP? (%)**					0.20
**Not at all familiar**	3.1	3	0	7.1	
**Somewhat familiar**	43.1	52.9	29.4	35.7	
**Very familiar**	53.8	44.1	70.6	57.2	
**Do you know enough about PrEP to have an informed discussion with patients?**^**a**^ **(% Yes)**	72.3	70.6	82.3	64.3	0.50
**Do you know where to refer a client to a PrEP provider in Philadelphia?**^**a**^ **(% Yes)**	81.5	70.6	94.1	92.9	0.08
***Objective questions with True/False answers***					
**PrEP is a way to prevent HIV by taking HIV treatment medication after exposure to the virus.**^**b**^ **(*Ans*: *False*; % Correct)**	72.3	61.8	82.3	85.7	0.20
**PrEP has been shown to work for gay men and transgender women.**^**b**^ **(*Ans*: *True*;** **% Correct)**	92.3	91.2	94.1	92.9	1.00
**PrEP is intended to be used by HIV positive (+) people.**^**b**^ **(*Ans*: *False*; % Correct)**	86.2	85.3	88.2	85.7	1.00
**The Food and Drug Administration has approved daily use of Truvada to reduce the risk of acquiring HIV.**^**b**^ **(*Ans*: *True*; % Correct)**	90.6	90.9	88.2	92.9	1.00
**Mean score for each provider (± SD)** **(Scale Range 0–4)**	3.4 ± 0.83	3.3 ± 0.86	3.5 ± 0.87	3.6 ± 0.65	0.20

N, number in that group; PrEP, Pre-exposure prophylaxis for HIV; SD, Standard Deviation.

^a^Dichotomous questions with yes/ no choices

^b^Dichotomous questions with true/false choices

^c^Others include non-tester, non-case manager providers or providers with multiple job roles

^d^ P-value shows any difference between case-managers and HIV testers based on Chi-square/ Fisher’s exact test/ Mann-Whitney U test statistics.

#### Knowledge

Overall, 97% of participants had previously heard of PrEP, but only 60% had been trained in screening clients for PrEP eligibility, and 46% reported not being very familiar with PrEP. On a 0–4 scale, the average knowledge score was 3.4 (SD = 0.83; Table 2).

#### Attitudes

Majority of the providers reported that they had adequate time to discuss PrEP (95%) and felt comfortable referring clients to PrEP providers (92%). Most of them thought that PrEP should be widely available to their clients (99%), that clients would be willing to adhere to PrEP (78%) and that PrEP screening should be part of routine HIV testing (97%). However, providers also reported concerns regarding stigma with PrEP use (74%) and the potential for increase in sexually transmitted diseases (64%), anti-retroviral medication resistance (52%), and PrEP doing more harm than good if not carefully implemented (33%). Important differences in attitudes were found between case managers and rapid HIV testers, with rapid HIV testers reporting more PrEP-related concerns, compared to case managers, particularly concerns regarding the potential to shift the focus and funding from other HIV prevention strategies and PrEP doing more harm than good. (Table 3).

**Table 3:** Attitudes of non-prescribing providers in Philadelphia (N = 66).

*Attitude Questions*	Total (N = 66)	Case managers (N = 34)	HIV testers (N = 18)	Other^b^ (N = 14)	P-value^c^
**PrEP should be widely available based on current evidence.**^**a**^ **(% Yes)**	98.5	100	100	92.9	-
**PrEP screening should be a part of the rapid HIV testing process** ^**a**^ **(% Yes)**	96.9	97	100	92.9	1.00
**I have the time to discuss PrEP as a part of preventative counseling.** ^**a**^ **(% Yes)**	95.2	97	88	100	0.30
**I feel comfortable referring a patient to a PrEP provider.** ^**a**^ **(% Yes)**	92.3	94.1	94.1	85.7	1.00
**Clients would be willing to adhere to PrEP daily.** ^**a**^ **(% Yes)**	77.8	81.3	76.5	71.4	0.70
**There will be little impact of PrEP on ARV resistance.** **(% Yes)**	47.5	48.4	47.1	46.2	0.90
**PrEP will not lead to an increase in STIs.** ^**a**^ **(% Yes)**	35.9	39.4	23.5	42.9	0.40
**PrEP could shift too much focus from other HIV prevention efforts.** ^**a**^ **(% Yes)**	27.0	18.2	47.1	23.1	0.03
**PrEP has the potential to do more harm than good if not carefully implemented.** ^**a**^ **(% Yes)**	33.3	24.2	52.9	30.8	0.04
**There is stigma for patients on PrEP.** ^**a**^ **(% Yes)**	74.2	65.6	70.6	100	0.70
**Funding for PrEP will reduce funding for other HIV preventions strategies.** ^**a**^ **(% Yes)**	21.9	12.1	41.2	21.4	0.03

PrEP, Pre-exposure prophylaxis for HIV; ARV, Antiretroviral (drugs); STIs, Sexually Transmitted Infections.

^a^Dichotomous questions with yes/ no choices

^b^Others include non-tester, non-case manager providers or providers with multiple job roles

^c^P-value shows any difference between case-managers and HIV testers based on Chi-square/ Fisher’s exact test statistics.

### Qualitative interviews

The characteristics of the subsample who completed qualitative interviews are shown in Table 1. The main themes emerging from the qualitative interviews and quotes illustrating the providers’ views are shown in Table 4.

**Table 4:** Themes emerging from the qualitative interviews.

Main Themes	Main Findings	Participant Quotes
**Attitude about PrEP: Positive views alongside serious concerns**	Most providers had positive views about PrEP and its effectiveness, and believed that their clients must be educated about PrEP.	*PrEP is*, *is wonderful*. *I'm glad that it’s out there*. *I'm going to keep referring it*, *you know*, *[…*.*] everyone should*, *you know*, *get more educated*.
	Some of the main concerns expressed by 33.3% of the providers included potential increase in sexually transmitted diseases, and limited access to PrEP, in the case of lack of insurance, financial instability, and homelessness.	*There's also a surge of chemsex [ph]*. *People are taking PrEP*, *taking meth*, *having unprotected sex with multiple partners sometimes at the same time*. *You will find yourself in a position where it's possible to proliferate*, *other infections*. *We've got a pretty big uptake in Philadelphia in Syphilis for example*.
		*If I know that [a] client is really unstable I wouldn’t recommend that they get on this medication that they need to take every day because there’s a great chance that they’ll fail*, *like they’ll have poor adherence*. *So*, *yeah*, *that will be a person that well*, *maybe*, *[I] wouldn’t necessarily push to take part*.
**Subjective Norms Regarding PrEP: Client Preferences and Organizational Support**	Providers perceived they had a short window of opportunity to interact with their clients, such as in the case of homeless IDUs encountered through street outreach.	*We have a site that’s called under the bridge and*, *all they do is*, *is shoot up there and they live under there so*, *of course*, *they want to come in and get out*, *you know*, *they don't want to be wasting too much time*. *They don't want to do (or know) more*, *you don't want to keep them in there more than what they want*, *because they get anxious*.
	Receiving support from coworkers and the organization, being able to share their experiences with their coworkers, and undergoing regular trainings boosted providers’ confidence and had a positive impact on the PrEP referral practices.	*But within our organization it's not that we're not encouraged to [refer people to PrEP]*. *It's quite the opposite*. *We have had Gilead come to our organization to speak with our staff and clients about PrEP*. *We’ve attended AACO [AIDS Activities Coordinating Office at the City of Philadelphia]*. *So*, *they run trainings as well and they do a great job of bringing providers in that will answer questions about the option [PrEP]*.
**Perceived Behavioral Control**	Some of the providers mentioned that they do not have sufficient time to talk to and answer all the queries of their clients, especially when the HIV testers have to meet a certain testing quota. In such circumstances, the providers do not initiate the conversation about PrEP.	*I mean we have a certain amount of*, *we have a quota to meet yearly*. *So*, *we sometimes do get bombarded with a lot of clients trying to assess them*. *So*, *like I said*, *we’ll [only] give them a quick overview*.
	Lack of availability of PrEP services was also found to be one of the barriers to PrEP referral. Under such circumstances, when clients are referred for PrEP, the providers fear that their clients may not act upon their initial interest or intent.	*Yeah*, *for one*, *we are a small organization*, *we are not like a clinic*, *so having an in-house I think would be beneficial*. *I think*, *um* .* *.* *. *yeah*, *anytime there is like multiple steps*, *I think there is always a possibility [of clients getting lost]*. *So*, *I think it would be much different if we had testing*, *counseling*, *referral and*, *like*, *‘go upstairs and get it*,*’ right in the same building*
	In organizations that have PrEP services on-site, we found that a limited number of physicians available to further assess the risk of the client and prescribe PrEP was one of the main barriers.	*We have the limitation that we can just only refer clients on Wednesdays mornings and Thursday*. *We only have a two day-limit for the client to come and see the doctor*, *so that limits our interaction and accessibility for them to get PrEP*.

PrEP, Pre-exposure prophylaxis for HIV; IDUs, intravenous drug users.

#### Attitudes about PrEP: Positive views alongside serious concerns

In general, providers viewed PrEP as a good prevention measure to reduce the burden of HIV. They were confident about the effectiveness of PrEP and believed that the public has to be educated about PrEP. Yet, some providers in the study (33.3%) also voiced personal concerns regarding PrEP, including worries about risk compensation behavior, lack of access to PrEP services, and lack of adherence to PrEP.

One of the main concerns was the potential for risk compensation behavior. Also known as behavioral disinhibition, risk compensation refers to the idea that risk practices will increase among PrEP users because of a perception of lowered risk conferred by the use of PrEP [31]. Providers in our study worried that PrEP users could believe PrEP is an “end-all” means to prevent HIV and stop using condoms and other STI preventative measures. Limited access to PrEP was also identified as one of the major barriers to more frequently refer at-risk clients for PrEP. Access in this context includes lack of insurance, financial instability, and homelessness. Providers mentioned that many of their clients are disenfranchised and do not have the means to afford PrEP or have other more pressing needs that must be met, like shelter and food. In those situations, they reported refraining from referring these clients to PrEP services and focusing on helping clients access other more urgently needed services.

#### Subjective norms regarding PrEP: Client preferences and organizational support

Providers in our study described their practices as “client-centered” and, from their perspective, referral for PrEP was in large dependent on their perception of interest and the amount of time the client had. When clients expressed in the forefront that they were not interested in learning about PrEP, some providers refrained from referring them for PrEP services. Study participants reported certain clients (e.g. homeless and/or IDUs encountered through street outreach) were often weary of being held for too long. In these circumstances, providers also refrained from initiating a conversation about PrEP.

Getting support from coworkers and being able to share experiences about PrEP was described by providers as having a positive impact on their PrEP referral practices. Regardless of their experiences, providers indicated they learn from each other and find validation for their PrEP referral practices through ongoing conversations with colleagues. They also reported overall encouragement and support for PrEP referrals from their organization, particularly in the form of trainings. In their opinion, continued education boosted providers’ confidence about talking to clients about PrEP and offered extra assurance that their organization was supporting them.

#### Perceived behavioral control: Constrains over decision to refer clients to PrEP

Lack of time severely diminished opportunities to engage in PrEP counseling on a regular basis. Some providers, especially HIV testers, explained they sometimes have only 10–15 minutes with each client, depending on the type of HIV testing kit used. Furthermore, in certain organizations, the HIV testers were required to meet testing goals (i.e. a certain number of clients tested over a certain period), which increased pressure to see more clients in less time and made providers hesitant to engage in conversations about PrEP.

Lack of availability of PrEP prescription services or limited availability of PrEP providers within their organization was also found to be a potential barrier to referring for PrEP. Providers in organizations that did not offer PrEP feared that high-risk clients may not act upon their initial interest when PrEP is not offered on-site. Even when PrEP prescription resources were available within the same organization, providers felt that there were few physicians who could prescribe PrEP to clients and their availability was limited to a few designated days a week (2–3 days). Both scenarios led to missing opportunity to link clients to PrEP services.

## Discussion

We evaluated PrEP-related knowledge, attitudes and referral practices of non-prescribing providers involved in HIV prevention using a combination of quantitative and qualitative methods. We found that only half of providers in our study reported that screening for PrEP was part of the rapid HIV testing process at their organization and 31% reported that eligible individuals were not referred for PrEP services. Only 1 in 4 indicated to personally screen their clients for PrEP eligibility and a mere 16% had referred more than five PrEP eligible candidates in previous 3 months. Yet, most (over 2 out of 3) reported having been asked about PrEP by clients. These figures indicate opportunity for these HIV prevention organizations to identify and link individuals who may benefit from PrEP, instead of placing the onus of learning about PrEP on clients. It has been reported that around 25% of men who have sex with men (MSM), 19% of intravenous drug users and 0.4% of heterosexually active adults in the USA are PrEP eligible [32], many of whom regularly interact with the non-prescribing providers such as the participants in our study. With right interventions, the non-prescribing providers are in a position to bring down these disparities.

Findings from our study yield insights into factors that may facilitate or impede referral of clients for PrEP by non-prescribing HIV providers. For example, our results reveal some gaps in knowledge about PrEP among our sample of providers. The vast majority (98%) of them had heard about PrEP, but 40% had not received formal training on this biomedical prevention option and almost half indicated they were not very familiar about PrEP. Compared to other studies about PrEP awareness among non-prescribing providers [18,19] and among doctors and nurses [33], our results are more positive, but they still point at the need to train front-line prevention providers to increase knowledge about PrEP, with special emphasis on HIV case managers, who demonstrated less PrEP-related awareness and self-confidence, as compared to HIV testers.

In our study, almost all providers felt that PrEP screening should be available and integrated into rapid HIV testing, but the data also suggested that some providers had doubts regarding PrEP implementation. Notably, our survey shows that the providers had concerns about stigma (74%), increased STIs (64%), anti-retroviral medication resistance (52%), and, to a lesser extent, about a shift in attention on (27%), and funding for (22%) other HIV prevention approaches. Front-line providers in previous studies had similar concerns (increased STIs, anti-retroviral medication resistance, and stigma), but the approval rate to widespread availability of PrEP was much lower (50 to 60%) [18,19]. Looking at provider types, referring >5 clients was more common in HIV testers than case managers. The former also showed higher levels of subjective knowledge, although no differences in objective knowledge were observed. Finally, a higher percentage of HIV testers than case managers had concerns about shift in focus and funding from other HIV prevention approaches, feasibility of discussing PrEP and potential for harm. These findings illustrate that different providers can have unique needs regarding knowledge and attitudes and they may be at different stages with regard to PrEP referrals. This information, if confirmed by future research, could inform the content of future interventions targeting different types of providers.

Data from the qualitative interviews supported and expanded the findings from our quantitative survey and were consistent with data published from studies conducted with HIV care providers and primary care providers (PCPs). In general, the qualitative data corroborated providers have mixed feelings about PrEP, with both positive views of the availability of this option and serious concerns about the potential impact on STI transmission and treatment resistance. Similar to research with other types of HIV providers, participants in our study shared a positive opinion about PrEP and believed the public must know about this prevention option [34]. Concerns about lack of adherence and unintended consequences of PrEP expressed by providers in our qualitative interviews also echoed findings from our survey and from previous research with providers of HIV care and PCPs [34, 35]. While research suggests attitudes toward PrEP and PrEP-related risk compensation are becoming more positive among providers [34], it is important to consider the reasons behind persistent negative concerns that we found in our study and address them in future PrEP training programs targeting front-line HIV prevention workers. Adding to findings from our survey, our qualitative study revealed the influence of interpersonal and organizational factors on PrEP-related practices. Client characteristics, attitudes and behavior affected providers’ decision to discuss PrEP. This is consistent with findings from previous research showing that client sexual orientation [36] and perceived client preferences influences provider decisions about whether or not PrEP should be initiated [34]. While such an approach reflects respect for client preferences, it can also result in exclusion from PrEP discussions of at-risk individuals who could benefit most from PrEP and, ultimately, exacerbate disparities in PrEP awareness and uptake. Since public awareness about PrEP still remains relatively low [37], it is vital for those involved in HIV prevention to educate and screen all clients and refer all those who are eligible for PrEP services.

Our qualitative data also underscore that peer norms and organization policies can play an important role in supporting providers’ practices by offering PrEP training and continued education, encouraging and providing peer-to-peer support activities, and designing services with PrEP in mind. Receiving support from coworkers and being able to confer with them when they had questions about PrEP were reported as facilitating PrEP referral practices. Other studies have highlighted that being able to share opinions and practices about PrEP with trusted colleagues has a positive influence on providers’ practices [35]. Providers reported that encouragement and support from their organizations in the form of continued education and trainings on PrEP boosted their confidence on talking about PrEP with their clients. This theme was consistent with previous research showing HIV providers want to have more knowledge and have a desire for trainings to more effectively promote PrEP [34]. Despite their best intentions, providers in our study also faced implementation barriers such as lack of time to educate patients about PrEP and screen for eligibility. The use of educational pamphlets about PrEP and inviting clients to return for follow-up discussion, if interested, could help to overcome some of the time constraints reported by prevention providers. Incorporating PrEP screening in the workflow of the institution could result in increased referrals for PrEP. This intervention can be particularly effective as only 50% of the participants reported that their institution routinely screens for PrEP, showing that there is room for improvement in institutional support to PrEP screening. This can be further incentivized by setting up a quota, similar to rapid HIV testing. Additionally, on-site provision of PrEP prescribing services can boost referrals. Our study found that only 14% of participants worked in settings where PrEP was provided on-site. The lack or limited availability PrEP prescribing services on-site discouraged some providers from referring clients to PrEP for fear they would not follow up on the referral if they had to go somewhere else or come back at another time.

To date, there have been few studies that have evaluated PrEP knowledge, attitude and practices among non- prescribing providers, who are often the first contact for those at high risk for HIV infection. Our study offers valuable tips for the design of training programs and structural interventions derived from the current attitudes and referral practices of non-prescribing providers in HIV prevention as well as from qualitative study of their perceptions and concerns to promote greater integration of PrEP screening and referrals into other HIV prevention services. Such trainings should involve rectifying inaccurate assumptions and unfounded concerns, while highlighting the effectiveness of PrEP and financial assistance programs that would increase client’s access to PrEP by making it affordable to them. In addition, as a result of PrEP outreach by non-prescribing providers, some of the burden of PrEP services on the prescribing providers will be alleviated giving them more time to educate and reinforcing the intervention.

## Limitations

There are a few study limitations. First, the prevalence of HIV is higher in Philadelphia, in comparison to other areas, and the findings in our study may not be generalizable to other lower prevalence cities or rural areas. Second, survey findings are based on a convenience sample of providers and qualitative data are restricted to a purposive subsample of participants. The study site and the sampling procedures may limit the generalizability of the findings to providers in other lower-prevalence and rural areas, as well as to all non-prescribing providers in Philadelphia. Thirdly, the study instruments were developed ad hoc for this study, although based on previous research, and the data are all based on self-report. These methods may compromise the validity of the findings. Finally, the small sample size limits the power of the quantitative study. Likewise, the small sample size of the qualitative component and the slightly higher education level of the providers interviewed represent an important limitation. Although we did not achieve data saturation (i.e. additional data may have uncovered new categories), the data available was sufficient to achieve theoretical saturation (i.e. it was sufficient to illustrate the constructs of the Theory of Planned Behavior) [38]. The interviews revealed relevant examples of attitudes, perceptions, and practices related to PrEP referral that shed some light on a rarely studied population of providers. These qualitative data add depth to the quantitative findings. The difference in education levels between the quantitative and qualitative samples could have resulted in findings that do not represent how providers with lower education levels feel about the subject matter. For all the reasons above, the results will need to be replicated by future studies with additional and larger samples of non-prescribing HIV prevention providers.

## Conclusion

Non-prescribing providers involved in HIV prevention are well aware and relatively knowledgeable about PrEP; yet, only 50% reported that screening for PrEP was part of the HIV testing process at their organization and only 16% of participants reported referring more than 5 clients for PrEP in the previous 3 months. Because these providers are on the front line of HIV prevention efforts, they could have opportunities to refer more clients for PrEP. Qualitative interviews found mixed attitudes about PrEP and underscored the role of social norms and organizational factors. Among the identified barriers were lack of time to discuss PrEP with clients, perceived lack of interest among client, and lack of access to a PrEP facility. To increase PrEP referrals from front line providers involved in HIV prevention, more providers need to be trained about PrEP, PrEP screening needs to be incorporated into the HIV testing process, and PrEP prescribing services should be offered on-site.

## Supporting information

S1 Appendix PrEP provider survey questionnaire. (DOCX)

S2 Appendix Factors influencing PrEP referral practices among HIV providers in Philadelphia qualitative study interview guide. (DOCX)

S1 Dataset PrEP provider quantitative survey dataset. (XLSX)

S2 Dataset Factors influencing PrEP referral practices among HIV providers in Philadelphia qualitative study dataset. (XLSX)

S1 Supporting Information PrEP provider quantitative survey data dictionary. (XLSX)
