# Supplementary material for: Attitudes and referral practices for pre-exposure prophylaxis (PrEP) among HIV rapid testers and case managers in Philadelphia: A mixed methods study
Source: PLoS One. 2019 Oct 7;14(10):e0223486. doi: 10.1371/journal.pone.0223486 (PMC6779237; doi:10.1371/journal.pone.0223486)
Supplement: S1 Appendix — (DOCX) [file pone.0223486.s001.docx]

PrEP Provider Survey

Thank you for taking the time to participate in our survey. Please answer each question to the best of your ability. The survey should take about 10 minutes to complete.

We are going to begin by asking some general questions.

1. What is your age?

Ans:

2. Is English your first language?

- Yes (0)
- No (1)

3. If No, what is your first language?

Ans:

1. What is your ethnicity? Choose One

- Non-Hispanic/ Non-Latino (0)
- Hispanic/Latino (1)

1. What is your race? (Check all that apply)

- African-American/ Black (0)
- Hispanic/ Latino (1)
- White (2)
- Asian (3)
- Native American (4)
- Native Hawaiian or other Pacific Islander (5)
- Other (6)

1. How do you identify yourself?

- Male (0)
- Female (1)
- I am a Trans man (2)
- I am a Trans woman (3)
- Other: (Please describe) (4)

1. What is the highest grade in school you completed?

- Some high school (0)
- High school diploma (1)
- Some college (2)
- Associates Degree (3)
- Bachelors Degree (4)
- Masters Degree (5)
- PhD (6)

1. Are you a non-prescribing provider, a case manager, or a navigator? Check all that apply

- Non-prescribing provider (0)
- Case manager (1)
- Navigator (2)
- HIV tester (3)
- Other : ___________________ (4)

1. How long have you been working in HIV prevention (years)?

Ans:

1. In what type of setting do you predominantly do HIV prevention work? (Check all that apply)

- Private practice (0)
- Community hospital (1)
- Academic hospital (2)
- Community health center (3)
- Walk in clinic (4)
- Sexual health clinic (5)

1. Does your practice serve a substantial population of any of the following (check all that apply)?

- Heterosexual Men and Women (0)
- MSM (1)
- Trans Women/ Men (2)
- IVDA (3)
- Prison inmate (4)
- Sex workers and their clients (5)

1. Is PrEP screening a part of rapid HIV testing process at your institution?

- Yes (0)
- No (1)
- Don’t know (2)

1. Do you personally screen clients who test HIV negative for PREP?

- Yes (0)
- No (1)

1. Is everyone who is eligible referred to PrEP services?

- Yes (0)
- No (1)

1. How many times were you asked about PrEP by clients in the previous 3 months?

- 0 (0)
- 1-5 (1)
- 6-10 (2)
- 11-20 (3)
- 21-50 (4)
- >50 (5)

1. How many people have you referred for PrEP in the previous 3 months?

- 0 (0)
- 1-5 (1)
- 6-10 (2)
- 11-20 (3)
- 21-50 (4)
- >50 (5)

**The following questions are to assess your knowledge regarding PrEP.**

1. Have you heard about PrEP before today?

- Yes (0)
- No (1)

1. If Yes, where have you heard about PrEP? Check all that apply

- Workshop, lecture or seminar (0)
- Colleague (1)
- Client/ Patient (2)
- Peer-reviewed medical journal (3)
- HIV/AIDS-related or other medical conference (4)
- Newspaper or magazine (5)
- Website or blog (6)
- Other, please specify (7)

1. Have you ever had formal training on PrEP?

- Yes (0)
- No (1)

1. How would you describe your current knowledge about PrEP?

- Not at all familiar (1st time hearing about it) (0)
- Somewhat familiar (I am aware of Prep but I am unaware of the details eligibility criteria) (1)
- Very familiar (I am aware of the details of eligibility criteria) (2)

1. Do you know enough about PrEP to have an informed discussion with patients?*

- Yes (0)
- No (1)

1. Do you know where to refer a client to a PrEP provider in Philadelphia?

- Yes (0)
- No (1)

**For the following six questions, please answer as True or False.**

1. PrEP is a way to prevent HIV by taking HIV treatment medication after exposure to the virus.

- True (0)
- False (1)

1. PrEP has been shown to work for gay men and transgender women.

- True (0)
- False (1)

1. PrEP is intended to be used by HIV positive (+) people.

- True (0)
- False (1)

1. Researchers do not know whether there are health risks from using PrEP for long periods of time. ***

- True (0)
- False (1)

1. The primary goal of treatment as prevention research is to test the safety of newly developed HIV medications. ***

- True (0)
- False (1)

1. The Food and Drug Administration has approved daily use of Truvada to reduce the risk of acquiring HIV.

- True (0)
- False (1)

**The following questions relate to your opinion about PrEP. There are no right or wrong answers, we just want to know your opinion.**

1. PrEP should be easily accessible based on current evidence.

- Yes (0)
- No (1)

1. I feel comfortable referring a patient to a PrEP provider.

- Yes (0)
- No (1)

1. Clients would be able to adhere to PrEP daily

- Yes (0)
- No (1)

1. There is no risk of physical or sexual coercion on PrEP

- Yes (0)
- No (1)

1. I am concerned that funding for PrEP will reduce funding for other HIV preventions

strategies

- Yes (0)
- No (1)

1. I have the time to discuss PrEP as a part of preventative counseling

- Yes (0)
- No (1)

1. PrEP is an effective prevention tool in the real world.

- Yes (0)
- No (1)

1. PrEP is more effective than PEP (post-exposure prophylaxis) for frequent PEP users.

- Yes (0)
- No (1)

1. PrEP will have greater impact than behavioral interventions on preventing HIV infection.

- Yes (0)
- No (1)

1. There is stigma for patients on PrEP

- Yes (0)
- No (1)

1. There is support from the gay community and gay press for PrEP.

- Yes (0)
- No (1)

1. Patients on PrEP will be perceived to be HIV positive by their peers

- Yes (0)
- No (1)

1. PrEP will have a significant impact on the HIV epidemic

- Yes (0)
- No (1)

1. Truvada is a safe drug for PrEP.

- Yes (0)
- No (1)

1. PrEP will not lead to an increase in STIs.

- Yes (0)
- No (1)

1. There will be little impact of PrEP on Anti-retroviral drug resistance.

- Yes (0)
- No (1)

1. There is not enough evidence available to justify making PrEP widely available in the US.

- Yes (0)
- No (1)

1. Investing in PrEP would be an inappropriate use of healthcare resources.

- Yes (0)
- No (1)

1. Policy makers have an ethical obligation to make available any intervention that could decrease an individual's risk of becoming infected with HIV.

- Yes (0)
- No (1)

1. PrEP could shift too much focus from other HIV prevention efforts.

- Yes (0)
- No (1)

1. PrEP is an exciting new HIV prevention tool and should be made more widely available as soon as possible.

- Yes (0)
- No (1)

1. PrEP has the potential to do more harm than good if not carefully implemented.

- Yes (0)
- No (1)

1. PrEP may be useful but is not ready to be made more widely available.

- Yes (0)
- No (1)

1. PrEP is a useless distraction.

- Yes (0)
- No (1)

1. PrEP is dangerous and should not be pursued further.

- Yes (0)
- No (1)

1. Should PrEP screening be a part of the rapid HIV testing process?

- Yes (0)
- No (1)

**Thank you for completing that portion of the survey. Now you are going to have a brief 30 minute education session about PrEP, followed by a 10 minute question and answer session.**

**Thank you for participating in the PrEP education session. We are now going to ask some of the previous PrEP questions again.**

**The following questions are to assess your knowledge regarding PrEP.**

1. Have you heard about PrEP before today?

- Yes (0)
- No (1)

1. If Yes, where have you heard about PrEP? Check all that apply

- Workshop, lecture or seminar (0)
- Colleague (1)
- Client/ Patient (2)
- Peer-reviewed medical journal (3)
- HIV/AIDS-related or other medical conference (4)
- Newspaper or magazine (5)
- Website or blog (6)
- Other, please specify (7)

1. Have you ever had formal training on PrEP?

- Yes (0)
- No (1)

1. How would you describe your current knowledge about PrEP?

- Not at all familiar (1st time hearing about it) (0)
- Somewhat familiar (I am aware of Prep but I am unaware of the details eligibility criteria) (1)
- Very familiar (I am aware of the details of eligibility criteria) (2)

1. Do you know enough about PrEP to have an informed discussion with patients?*

- Yes (0)
- No (1)

1. Do you know where to refer a client to a PrEP provider in Philadelphia?

- Yes (0)
- No (1)

**For the following six questions, please answer as True or False.**

1. PrEP is a way to prevent HIV by taking HIV treatment medication after exposure to the virus.

- True (0)
- False (1)

1. PrEP has been shown to work for gay men and transgender women.

- True (0)
- False (1)

1. PrEP is intended to be used by HIV positive (+) people.

- True (0)
- False (1)

1. Researchers do not know whether there are health risks from using PrEP for long periods of time. ***

- True (0)
- False (1)

1. The primary goal of treatment as prevention research is to test the safety of newly developed HIV medications. ***

- True (0)
- False (1)

1. The Food and Drug Administration has approved daily use of Truvada to reduce the risk of acquiring HIV.

- True (0)
- False (1)

**The following questions relate to your opinion about PrEP. There are no right or wrong answers, we just want to know your opinion.**

1. PrEP should be easily accessible based on current evidence.

- Yes (0)
- No (1)

1. I feel comfortable referring a patient to a PrEP provider.

- Yes (0)
- No (1)

1. Clients would be able to adhere to PrEP daily

- Yes (0)
- No (1)

1. There is no risk of physical or sexual coercion on PrEP

- Yes (0)
- No (1)

1. I am concerned that funding for PrEP will reduce funding for other HIV preventions

strategies

- Yes (0)
- No (1)

1. I have the time to discuss PrEP as a part of preventative counseling

- Yes (0)
- No (1)

1. PrEP is an effective prevention tool in the real world.

- Yes (0)
- No (1)

1. PrEP is more effective than PEP (post-exposure prophylaxis) for frequent PEP users.

- Yes (0)
- No (1)

1. PrEP will have greater impact than behavioral interventions on preventing HIV infection.

- Yes (0)
- No (1)

1. There is stigma for patients on PrEP

- Yes (0)
- No (1)

1. There is support from the gay community and gay press for PrEP.

- Yes (0)
- No (1)

1. Patients on PrEP will be perceived to be HIV positive by their peers

- Yes (0)
- No (1)

1. PrEP will have a significant impact on the HIV epidemic

- Yes (0)
- No (1)

1. Truvada is a safe drug for PrEP.

- Yes (0)
- No (1)

1. PrEP will not lead to an increase in STIs.

- Yes (0)
- No (1)

1. There will be little impact of PrEP on Anti-retroviral drug resistance.

- Yes (0)
- No (1)

1. There is not enough evidence available to justify making PrEP widely available in the US.

- Yes (0)
- No (1)

1. Investing in PrEP would be an inappropriate use of healthcare resources.

- Yes (0)
- No (1)

1. Policy makers have an ethical obligation to make available any intervention that could decrease an individual's risk of becoming infected with HIV.

- Yes (0)
- No (1)

1. PrEP could shift too much focus from other HIV prevention efforts.

- Yes (0)
- No (1)

1. PrEP is an exciting new HIV prevention tool and should be made more widely available as soon as possible.

- Yes (0)
- No (1)

1. PrEP has the potential to do more harm than good if not carefully implemented.

- Yes (0)
- No (1)

1. PrEP may be useful but is not ready to be made more widely available.

- Yes (0)
- No (1)

1. PrEP is a useless distraction.

- Yes (0)
- No (1)

1. PrEP is dangerous and should not be pursued further.

- Yes (0)
- No (1)

1. Should PrEP screening be a part of the rapid HIV testing process?

- Yes (0)
- No (1)

**Thank you for taking the time to complete our survey.**
