# Supplementary material for: Attitudes and referral practices for pre-exposure prophylaxis (PrEP) among HIV rapid testers and case managers in Philadelphia: A mixed methods study
Source: PLoS One. 2019 Oct 7;14(10):e0223486. doi: 10.1371/journal.pone.0223486 (PMC6779237; doi:10.1371/journal.pone.0223486)
Supplement: S2 Appendix — (DOCX) [file pone.0223486.s002.docx]

**Factors Influencing PrEP Referral Practices Among HIV Providers in Philadelphia**

**Qualitative Study Interview Guide**

| Study # Participant ID:  Date: Role of the Participant:  Interviewer:  Location:  Start Time:  End Time: |
| --- |
| Opening Statements: Thank you for taking the time to talk with me. My name is Navya Karkada and I am part of the PrEP research team at Drexel University School of Public Health. You have been asked to participate in this interview because you had participated in a quantitative survey and attended an educational session on PrEP conducted by Dr. Zsofi Szep. We are talking to people who are working in the HIV prevention field to get a better understanding about their perceptions, attitudes, knowledge, and barriers and facilitators to discussing about PrEP with their clients and referring them to PrEP services.  Through this interview, I want to learn more about your thoughts, feelings and experiences working as a HIV provider and about PrEP. The interview will take about 45 minutes but if you need a break at any time, please let me know. Also, you are not obligated to answer all the questions that I ask. If you feel uncomfortable answering any questions, you do not have to answer them. You are under no obligation to complete the interview; we can stop the interview at any time.  You will receive a $10 gift card for completing the interview. The interview will be audio recorded so that I can recall what we discussed and use it for data analysis.  Do you have any questions? |

| Themes or Domains | Sample Prompt Question | Sample Follow-up Probes |
| --- | --- | --- |
| Participant Basic Work Information | Please describe what do you do on a typical day as a part of your job as an HIV provider. | - How many HIV positive clients do you see each day? - How many HIV negative clients do you see each day? - How do you assess high risk patients? - What kind of counseling do you provide to your clients? |
| PrEP Knowledge and Perception | Please tell me what you know about PrEP, its efficacy and uses. | - What do you know about the effectiveness of PrEP? - Who is PrEP indicated for? - Which groups of people, if any, should be referred to PrEP? - How effective do you think PrEP is in reducing the likelihood of contracting HIV? |
| PrEP Attitudes | What are your views on PrEP as a prevention option for at risk individuals? | - What are your reservations, if any, when it comes to referring individuals at high risk of contracting HIV to PrEP? - What are your views on the risk compensation or the idea that people may engage in more risk behaviors if they are on PrEP? - What are your views about the risk of developing resistance to ARV or the idea that some groups may not adhere to PrEP correctly? |
| Perceived Power | Tell me about your referral practices.  How often do your refer your HIV positive clients partners for PrEP and how do you go about it? | - How comfortable do you feel discussing about PrEP and referring PrEP to your clients? - Tell me about particular types of clients or circumstances that make it more or less likely for you to talk about PrEP or refer a client to PrEP. - Tell me about your experiences talking to your clients about PrEP and referring them to PrEP services. - How do conversations with clients impact your referral or intention of referral to PrEP? Please explain. - Tell me about the reactions of your clients when you talk to them about PrEP. - Can you think of any instances in which clients have refused to engage in a conversation about PrEP? Please, describe. - Can you think of instances in which clients appeared uncomfortable when you were talking about PrEP? Please, describe. - Do they seem to understand the information? What are some of the questions or concerns that the clients have expressed regarding PrEP? - Have you changed the way you approach a conversation about PrEP based on your past experiences? - How do conversations with clients impact your referral or intention of referral to PrEP? Please explain. |
| Barriers | If not, why don’t you refer your clients to PrEP?  (If respondent does refer clients to PrEP) Are there some circumstances when, or types of clients, you don’t refer to PrEP? Tell me more about those instances.  What are your concerns or barriers to offering PrEP to your clients? | - What are some of the barriers to referring PrEP? - Do you have some personal concerns or questions that stop you from referring clients to PrEP? - Are there factors in your organization or the nature of your job that make it hard for you to refer clients to PrEP? - In relation to service provision - In relation to client: acceptance   Any other reasons |
| Subjective Norms | What are the attitudes of your colleagues or organization about PrEP? | - Can you recount a conversation you had about PrEP with your colleagues? - What attitudes do the leaders in your organization have about PrEP? - How positive, encouraging or negative, discouraging have been your conversations with colleagues about PrEP referral? - How do leadership attitudes affect what you do or don’t do about PrEP? |
| Intention to refer PrEP | Going forward, how frequently do you think you will be referring clients to PrEP? Why?  How frequently do you think you will be referring your HIV positive clients’ partners to PrEP? | - (If not very frequently) How do you think that you can overcome some of the factors that hinders you from referring PrEP? - What barriers you think you will encounter and how do you think you could overcome them? |
| Information Session Evaluation | Before you attended the information session with Drs. Szep and Sandling, from where did you get your information about PrEP? | - What were you main sources of information about PrEP? (Probe for:   - Conferences or professional meetings   - Colleagues, Supervisors   - Other sources within your organization   - Trainings   - Internet |
| Information Session Evaluation | How has attending the information session on PrEP conducted by Drs. Szep and Sandling changed your referral practices? | - How many clients have you referred to PrEP since the information session in the past 3 months? How does this compare to your prior referral rates? - How many of those have followed up with a provider? - How many of the clients you referred PrEP to have started taking PrEP? |
| Information Session Evaluation | How has your perspective about PrEP changed since the information session? | - Has there been a difference in that way you talk to your clients about PrEP? If so, how is it different? |
| Information Session Evaluation | How would you recommend we change the information session to be more effective? | - What more would you want to learn? |
